# Preparation, Characterization and Application of Magnetic Fe_3_O_4_-CS for the Adsorption of Orange I from Aqueous Solutions

**DOI:** 10.1371/journal.pone.0108647

**Published:** 2014-10-01

**Authors:** Yankai Du, Meishan Pei, Youjun He, Faqi Yu, Wenjuan Guo, Luyan Wang

**Affiliations:** School of Chemistry and Chemical Engineering, University of Jinan, Jinan, Shandong Province, China; Indian Institute of Toxicology Reserach, India

## Abstract

Fe_3_O_4_ (Fe_3_O_4_-CS) coated with magnetic chitosan was prepared as an adsorbent for the removal of Orange I from aqueous solutions and characterized by FTIR, XRD, SEM, TEM and TGA measurements. The effects of pH, initial concentration and contact time on the adsorption of Orange I from aqueous solutions were investigated. The decoloration rate was higher than 94% in the initial concentration range of 50–150 mg L^−1^ at pH 2.0. The maximum adsorption amount was 183.2 mg g^−1^ and was obtained at an initial concentration of 400 mg L^−1^ at pH 2.0. The adsorption equilibrium was reached in 30 minutes, demonstrating that the obtained adsorbent has the potential for practical application. The equilibrium adsorption isotherm was analyzed by the Freundlich and Langmuir models, and the adsorption kinetics were analyzed by the pseudo-first-order and pseudo-second-order kinetic models. The higher linear correlation coefficients showed that the Langmuir model (R^2^ = 0.9995) and pseudo-second-order model (R^2^ = 0.9561) offered the better fits.

## Introduction

Currently, dyes are widely used in many industrial applications, including textiles, printing, plastics, food, leather and papermaking, among others [1]–[4]. In China, large amounts of dye wastewater are directly discharged into natural water courses without treatment, particularly in the rural area of China [5]. Wastewater containing dyes and dyed products has caused pollution in many areas [6]–[10]. Due to the complex structure of their aromatic molecules, most azo dyes biodegrade slowly if at all, which causes a natural unbalance in the environment [11]–[13]. Some dyes containing special compounds are considered toxic to both human and animals even at very low concentrations, generally most of these compounds cause mutagenic, teratogenic and carcinogenic effects which subsequently lead to the generation of health disorders such as dysfunction of the kidney, reproductive system, liver, brain, and central nervous system [14]. The improper management of industrial water containing dyes is a source of pollution.

Orange I belongs to the family of azo dyes, which represent around 50% of all dyes used in textile industry [15]–[16]. Because it contains an -N = N- chromophore group, Orange I is highly toxic and causes various diseases [17]–[18], such as nausea, carcinogen, dermatitis, methemoglobinemia, tumors and allergies [19]–[20]. Being anionic in nature, orange I has high potential to leach into the soil profile and to contaminate ground water [21]. Wastewater containing Orange I can seriously harm human health if discharged into freshwater. The orange coloration is also a type of environmental pollution [1]. Environmental restrictions have been established by many local governments to control the quality of colored effluents and force industries to decolorize their effluents before discharging them 22]. Therefore, it is necessary to remove Orange I from wastewater before it is discharged into bodies of freshwater.

Several methods of dye removal have been developed during the global environmental movement, including photocatalysis [23], coagulation [24], and adsorption [25]. Among the processes for treating colored wastewater, adsorption is the best choice [26]–[27], because it is a low cost and easy to implement method [28]. Normal adsorbents such as active carbon and alumina have been used to adsorb dyes in wastewater, but their adsorptive capacity is not as high as expected [1], and they are expensive. It is therefore necessary to find a cheaper and more effective adsorbent substitute for the normal adsorbents.

Chitosan (CS) is a natural biopolymer obtained from the process of alkaline deacetylation of chitin [29]. It is hydrophilic, biocompatible, biodegradable and antibacterial. Chitosan is an ideal adsorbent because of its functional groups. Each glucosamine unit has one amine group (–NH_2_) and two hydroxyl groups (−OH) [30]. Under acidic conditions, the amine groups of chitosan become protonated, and the positive group (−NH_3_^+^) can adsorb some negative ions through electrostatic interaction. The adsorption capacity of chitosan could also be strengthened by forming a hydrogen bond between the hydroxyl groups (−OH) and the adsorbed molecules. However, pure chitosan does not have optimal adsorption because it easily dissolves in acidic solution and has weak chemical resistance [31]. Blending chitosan with magnetic Fe_3_O_4_ can effectively avoid the chemical weakness of chitosan, the hydroxyl groups on the surface of Fe_3_O_4_ can interact with amine groups and hydroxyl groups of chitosan through hydrogen-bond interaction to keep chitosan stable under acidic condition. The obtained adsorbent is also easily prepared, inexpensive and has high adsorptive capacity. Compared with other magnetic adsorbents, the cost of the adsorbent prepared is about 1/2 of amino-functionalized silica-coated Fe_3_O_4_ [32] and 1/3 of amine-modified silica magnetite [33]. The magnetic adsorbent thus obtained was used to process wastewater containing the acid dye orange I.

Commonly, magnetic adsorbents are prepared by two-steps method [34]–[35], which has a complicated preparation process and low production. In this study, Fe_3_O_4_ (Fe_3_O_4_-CS) coated with magnetic chitosan was prepared by a one-step method. The resulting Fe_3_O_4_-CS was characterized by Fourier transform infrared spectroscopy (FTIR) and X-ray diffraction (XRD). The morphology of Fe_3_O_4_-CS was examined by scanning electron microscopy (SEM) and transmission electron microscopy (TEM). The chitosan content was measured by thermal gravimetric analysis (TGA). Orange I was adsorbed from an aqueous solution at room temperature (25°C), and the adsorbent could be easily be separated from the Orange I solution by magnetism. The effects of pH, initial concentration, and contact time were investigated. The adsorption isotherm and adsorption kinetics were studied for a comprehensive understanding of the adsorption process.

## Methods

### 2.1 Chemical and materials

Chitosan with a 95% degree of deacetylation, FeCl_3_ (97%) and FeSO_4_·7H_2_O (99%) were purchased from Sinopharm Chemical Reagent Co. Ltd. Orange I (C_16_H_11_N_2_NaO_4_S, MW = 350.32) was purchased from Aladdin Chemistry Co. Ltd. A stock solution was prepared by dissolving 0.4 g Orange I in 500 mL of distilled water, which was diluted to approximate concentrations. Other reagents used in this study were all analytical grade, and all solutions were prepared by using deionized water.

### 2.2 Preparation of magnetic Fe_3_O_4_-CS

First, 2.17 g of FeCl_3_ and 0.77 g of FeSO_4_·7H_2_O were dissolved in 50 mL of deionized water in a 250 mL flask. The mixture was vigorously stirred in a water bath at 313 K for 30 min. Then 0.35 g of chitosan was dissolved in 100 mL of 1% (v/v) acetic acid. The chitosan solution was added to the flask and vigorously stirred for 2.0 h. Then, 48 mL of NH_3_·H_2_O was added dropwise over 2.0 h, and the solution was vigorously stirred for another 1.0 h. Throughout the process, the temperature was maintained at 313 K, and the whole process was conducted under protection of N_2_ gas. After the reaction, the product was filtered and washed with distilled water and ethanol 3 times. Then, the precipitate was dried in a vacuum oven at 333 K. The obtained product was Fe_3_O_4_-CS.

### 2.3 Characterization of Fe_3_O_4_-CS

FTIR spectra of Fe_3_O_4_-CS were recorded on a Bruker VECTOR-22 IR spectrometer. KBr and the sample (approximately 1% mass of KBr) were mixed together, and then the mixture was pestled and pressed into a tablet. The spectra were collected over the spectral range of 400–4000 cm^−1^.

XRD patterns were recorded on a Rigaku D/max 2500 kV PC X-ray diffractometer operating at 40 Kv. The scan angle 2θ varied from 10° to 80° and the scan speed was 0.03° s^−1^.

SEM images were taken on a Quanta 200 (Philips-FEI, Holland). The SEM images were taken by applying 10 kV voltage with various magnification times for the observation of the surface.

TEM was obtained with a JEM-2100F microscope using an accelerating voltage of 200 kV. The samples were lightly ground and then dispersed ultrasonically in ethanol. A drop of the suspension was evaporated on a’holey’carbon film and pre-deposited on 200-mesh copper grids.

TGA were conducted on a Perkin–Elmer Diamond TG/DTA Instrument with a heating rate of 10°C min^−1^ under a nitrogen flow at temperatures ranging from 25 to 800°C. During the TGA measurement, the ratio of chitosan content in the Fe_3_O_4_-CS was obtained.

### 2.4 Batch adsorption experiments

First, 0.05 g of Fe_3_O_4_-CS and 40 mL of Orange I aqueous solution were added into a 50 mL conical flask. After agitation for 3 h at a rate of 225 rpm, the flask was placed on a magnet, and the Fe_3_O_4_-CS was aggregated on the bottom by magnetic force. One milliliter of supernatant was diluted in a 50 mL volumetric flask to obtain a measurable absorption. The concentration of the Orange I in the solution was immediately determined via UV-vis spectroscopy (Purkinje General, TU-1901) at an optimal wavelength of 476 nm, which corresponds to the maximum absorbance for Orange I. The adsorption ability was calculated using absorbance values measured before and after adsorption according to the following equation:
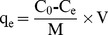
(1)Where q_e_ is the amount of dye adsorbed by the adsorbent (mg g^−1^), C_0_ is the initial dye concentration (mg L^−1^), C_e_ is the dye concentration after adsorption (mg L^−1^), M is the mass of Fe_3_O_4_-CS adsorbent (g), and V is the volume of dye solution (L).

All the adsorption experiments were conducted for 3 times and mean values were used as the experimental data to make the results reliable.

#### 2.4.1 Effect of pH

A series of conical flasks containing 40 mL of Orange I solution with an initial concentration of 400 mg L^−1^ was adjusted to a pH range of 1.0–9.0 using HCl (1 M) and NaOH (1 M) solutions. Then, 0.05 g of Fe_3_O_4_-CS was added to each flask, and the flasks were shaken for 3 h at room temperature.

#### 2.4.2 Effect of initial concentration

First, 0.05 g of Fe_3_O_4_-CS and 40 mL of Orange I solution with concentrations in the range of 50–800 mg L^−1^ was added to each of a series of conical flasks. The effect of the initial dye concentrations was studied after agitation for 3 h at the optimum pH value (pH 2.0).

#### 2.4.3 Effect of contact time

First, 0.05 g of Fe_3_O_4_-CS and 40 mL of Orange I solution with an initial concentration of 400 mg L^−1^ were added to each of a series of flasks labeled 1–12. The effect of contact time was analyzed after 5, 10, 15, 20, 25, 30, 60, 90, 120, 180, 240 and 300 minutes of shaking at the optimum pH value (pH 2.0), and the flasks were used for analyzing at different time intervals.

## Results and Discussion

### 3.1 Characterization of the Fe_3_O_4_-CS adsorbent

**Figure 1:**
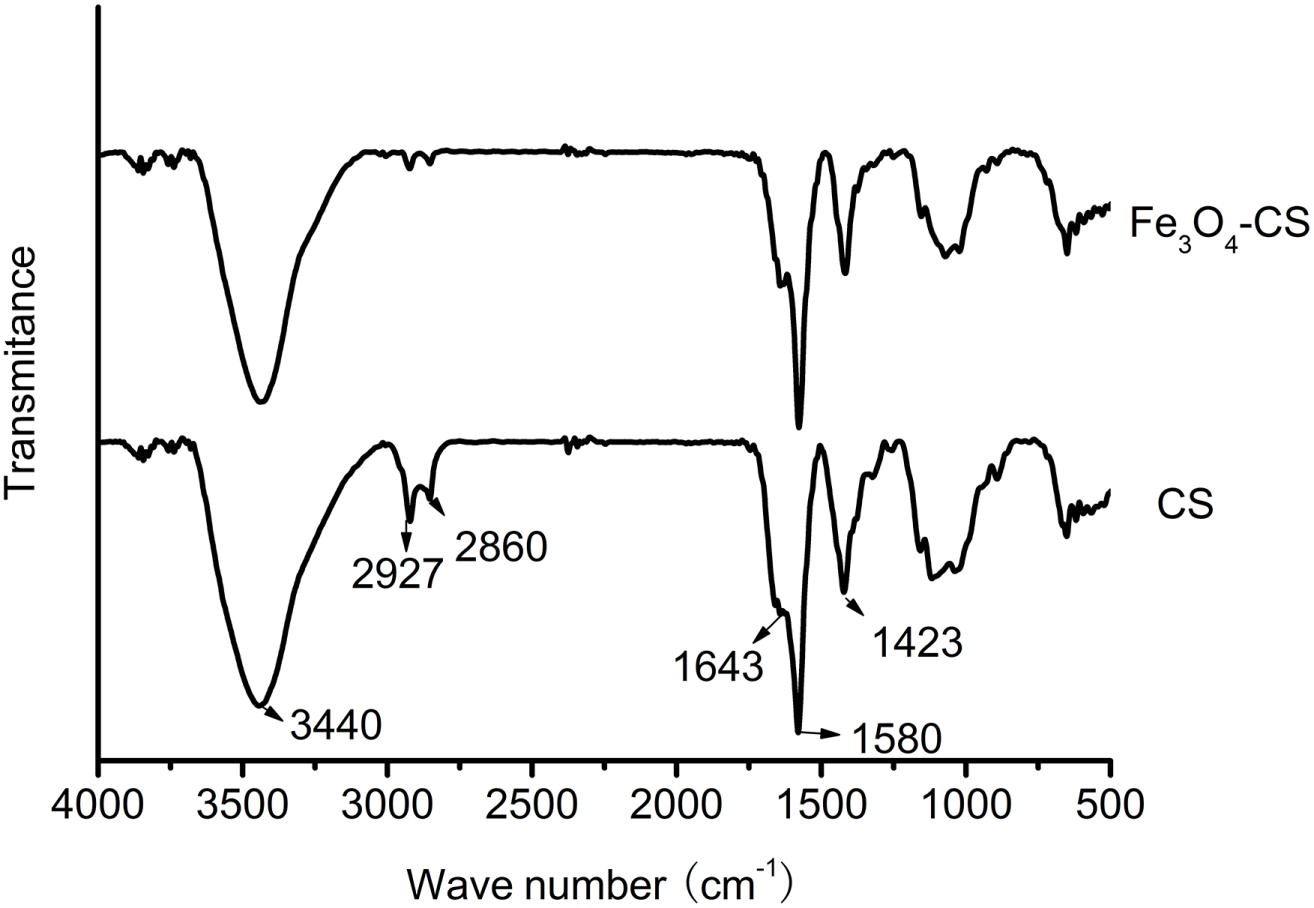
FTIR spectra of CS and Fe_3_O_4_-CS.

The FTIR spectra of chitosan and Fe_3_O_4_-CS are shown in Fig. 1. As shown, the spectra of Fe_3_O_4_-CS are almost consistent with the spectra of chitosan. The adsorption at approximately 3440 cm^−1^ reflects the overlapping of the stretching vibration of the O-H groups and N-H groups. The adsorption at 2927 cm^−1^ and 2860 cm^−1^ is due to the C-H stretching vibration of the -CH_2_ groups in chitosan. The adsorption at 1643 cm^−1^ is attributed to the deformation vibration of primary amine, the one at 1580 cm^−1^ is attributed to the N-H deformation vibration of -NH_2_ groups, and the one at 1423 cm^−1^ is attributed to the C-N stretching vibration. The FTIR spectra in Fig. 1 clearly demonstrate the existence of chitosan in the Fe_3_O_4_-CS.

**Figure 2:**
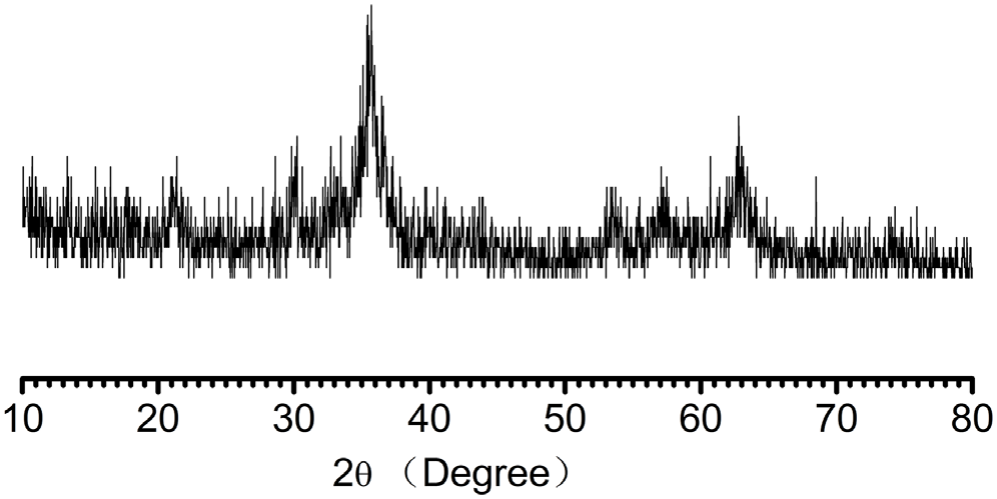
XRD patterns of Fe_3_O_4_-CS.

The XRD pattern of Fe_3_O_4_-CS is shown in Fig. 2. There are characteristic peaks at 2θ = 30.1°, 35.4°, 43.1°, 53.4°, 56.9° and 62.5°, which correspond to the (220), (311), (400), (422), (511) and (440) crystal planes of Fe_3_O_4,_ respectively; these peaks are consistent with the PDF card in the database (PDF No. 19-0629). This indicates the existence of Fe_3_O_4_, and the obtained adsorbent can be separated from aqueous solutions by magnets [36].

**Figure 3:**
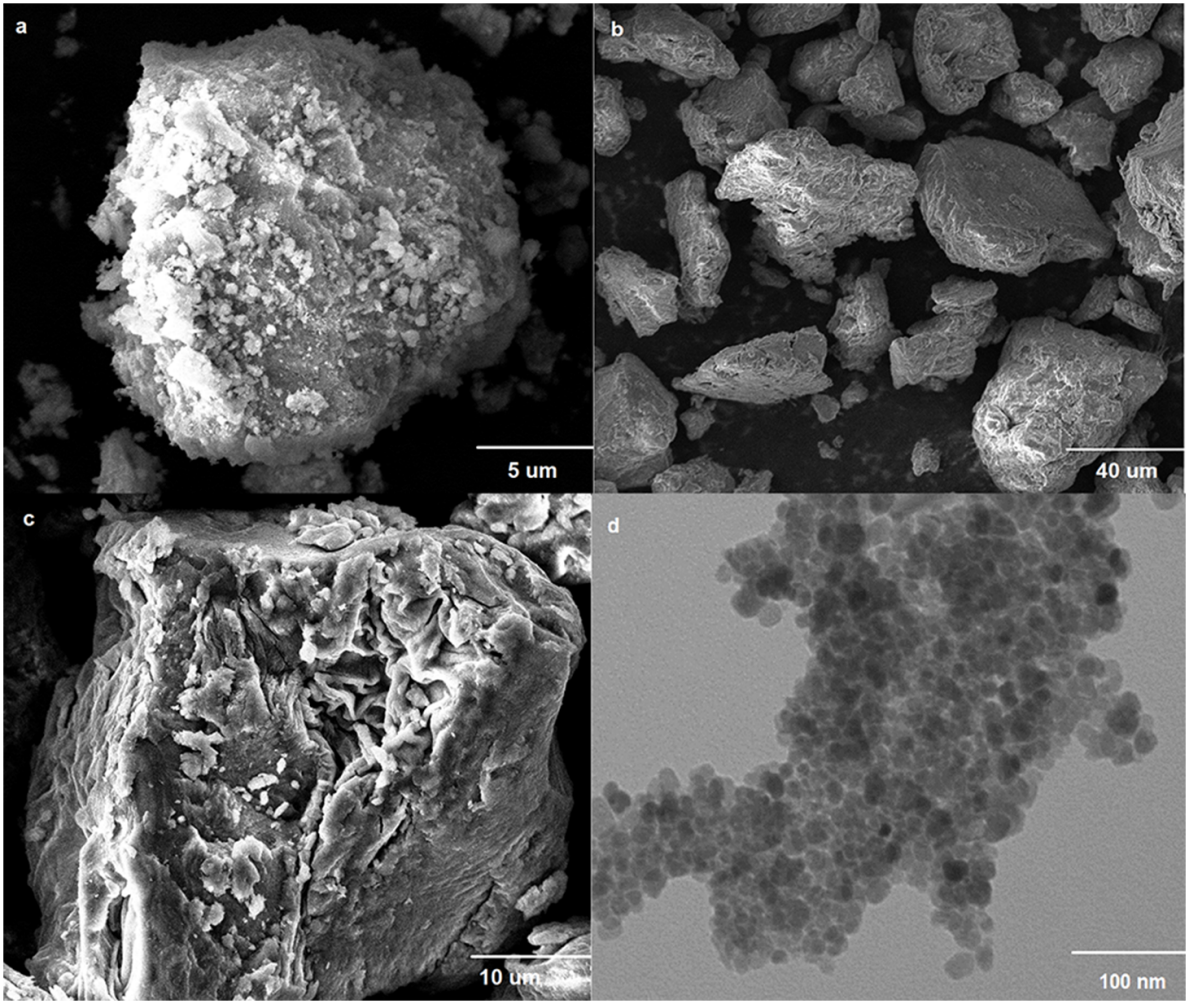
SEM images (a, b, c) and TEM image (d) of Fe_3_O_4_ and Fe_3_O_4_-CS.

**Figure 4:**
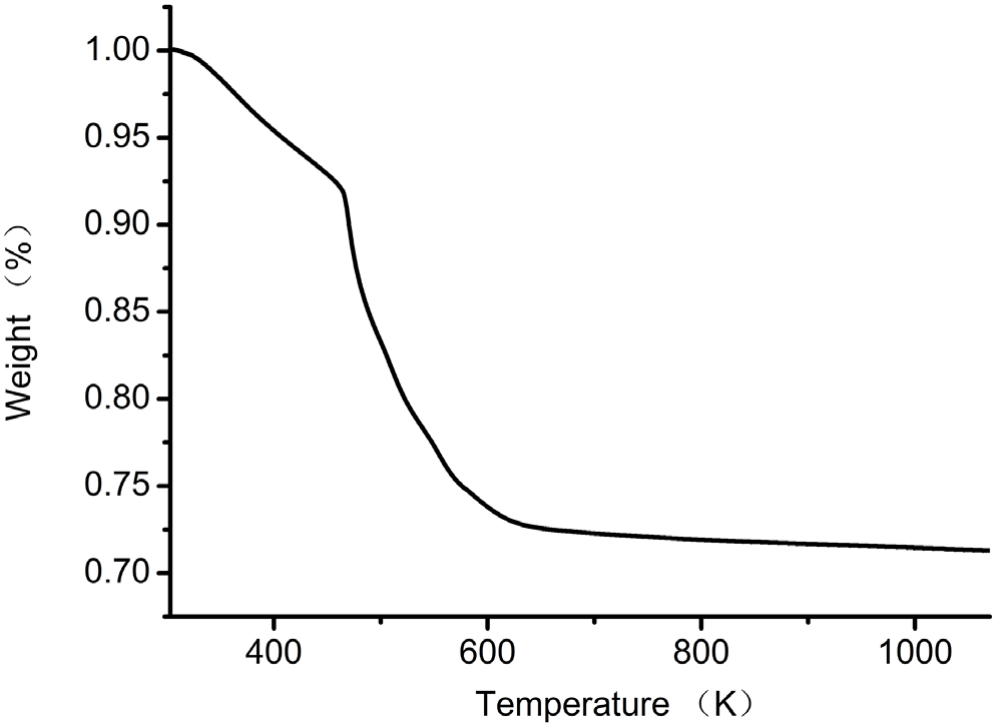
TGA curve of Fe_3_O_4_-CS.

Pure Fe_3_O_4_ was prepared in order to make a comparison with Fe_3_O_4_-CS on the surface structure. The surface structure of the pure Fe_3_O_4_ and synthesized magnetic Fe_3_O_4_-CS is shown in SEM images of Fig. 3. In Fig. 3.(a), the surface of pure Fe_3_O_4_ is rough and irregular. After being coated with chitosan, the surface of the resulting Fe_3_O_4_-CS becomes smooth, and the folding structure can be clearly observed in Fig. 3.(b). In Fig. 3.(c), the folds on the surface of the Fe_3_O_4_-CS adsorbent are more distinct. They are formed by the coating of organic chitosan on the surface of Fe_3_O_4_. Fig. 4.(d) is the TEM image of Fe_3_O_4_-CS. In Fig. 3.(d), the nanoparticles, which have a spherical structure and uniform particle size, could be observed. The color of the center of the sphere is darker, which is ascribed to the existence of Fe_3_O_4_. In contrast, due to the coating of organic chitosan, the color of the edge of the sphere is lighter. Through the SEM and TEM measurements, the microtopography of Fe_3_O_4_-CS could be clearly observed, and the nanoparticle size is approximately 20 nm.

Fig. 4 shows the TGA curve of Fe_3_O_4_-CS in the temperature range of 303 K-1000 K at a heating rate of 10 K min^−1^. The weight lost from the Fe_3_O_4_-CS adsorbent was divided into three different temperature ranges. A 6.5% loss in the first stage was ascribed to the loss of absorbed and bound water between 303 K (30°C) and 423 K (150°C). Approximately 20% of the weight loss in the temperature range occurred between 463 K (190°C) and 603 K (330°C) in the second stage, which was due to the degradation and deacetylation of chitosan. Approximately 1% of the weight loss was in the third stage, which was due to further degradation of chitosan at 653–773 K (380–500°C). The practical output of Fe_3_O_4_-CS was 1.40 g and the theoretical output of Fe_3_O_4_-CS was 1.60 g. According to the TGA analysis, water content in Fe_3_O_4_-CS was about 6.5%, then the pure Fe_3_O_4_-CS obtained was about 1.31 g, the productivity was about 81.8%, the high productivity make the adsorbent economical and practical.

### 3.2 Effect of pH

**Figure 5:**
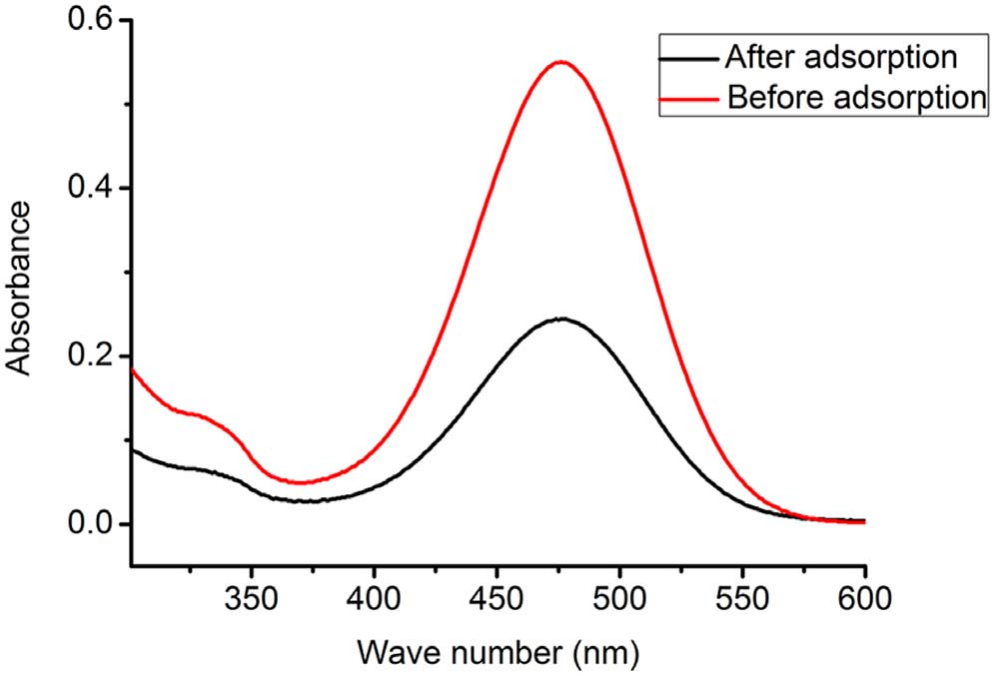
UV-vis spectra of Orange I solution before and after adsorption.

**Figure 6:**
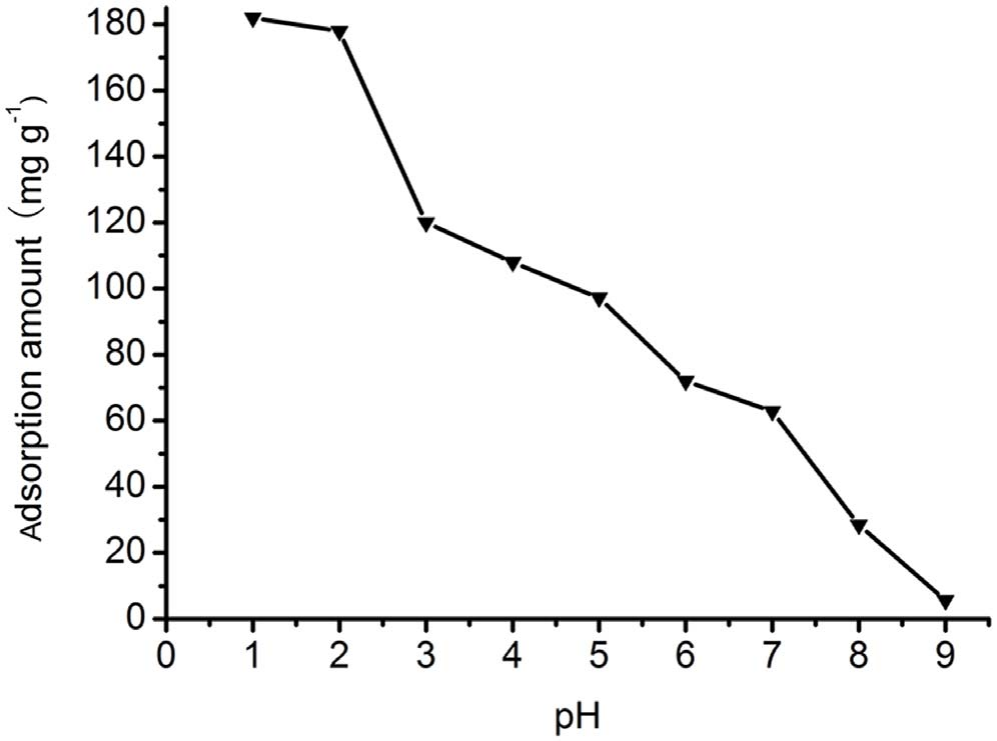
Effect of pH on Orange I adsorption by Fe_3_O_4_-CS.

**Figure 7:**
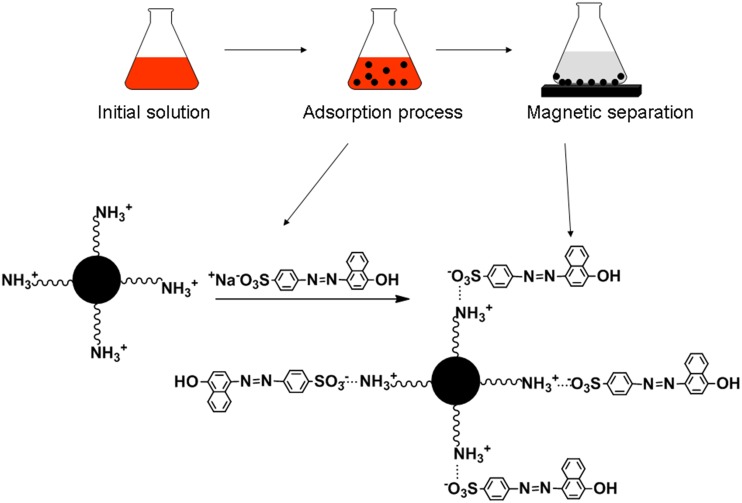
Schematic illustration for adsorption of Orange I by Fe_3_O_4_-CS.

UV-vis spectra of Orange I solution (400 mg L^−1^, pH 2.0) before and after adsorption is presented in Fig. 5. The absorbance after adsorption decreases sharply compared with absorbance before adsorption, which indicates a much lower concentration after adsorption. The effect of pH on adsorption is shown in Fig. 6. The adsorption amount decreases with increasing pH in the pH range from 1.0 to 9.0. The mechanism of adsorption relies on the ionic interactions between amino groups (–NH_3_^+^) and sulfonate groups (–SO_3_^−^) of Orange I (shown in Figure 7). At pH 2.0, the adsorption amount is 178 mg g^−1^, and it decreases to 5.6 mg g^−1^ at pH 9.0. At pH 1.0, the adsorption amount does not increase obviously compared with adsorption amount at pH 2.0. This can be attributed to the protonation of the –NH_2_ groups into –NH_3_^+^ in the presence of H^+^ ions under acidic conditions. At pH 2.0, the –NH_2_ groups were already completely protonated into –NH_3_^+^, and the electrostatic interaction between –NH_3_^+^ and -SO_3_^−^ was strengthened. With increasing pH, the amount of –NH_3_^+^ decreased, and when the pH passes the isoelectric point of chitosan, the –NH_3_^+^ groups deprotonated to the form of –NH_2_ groups, and the electrostatic interaction disappeared. Therefore, the optimal pH of adsorption is 2.0.

### 3.3 Effect of initial concentration

**Figure 8:**
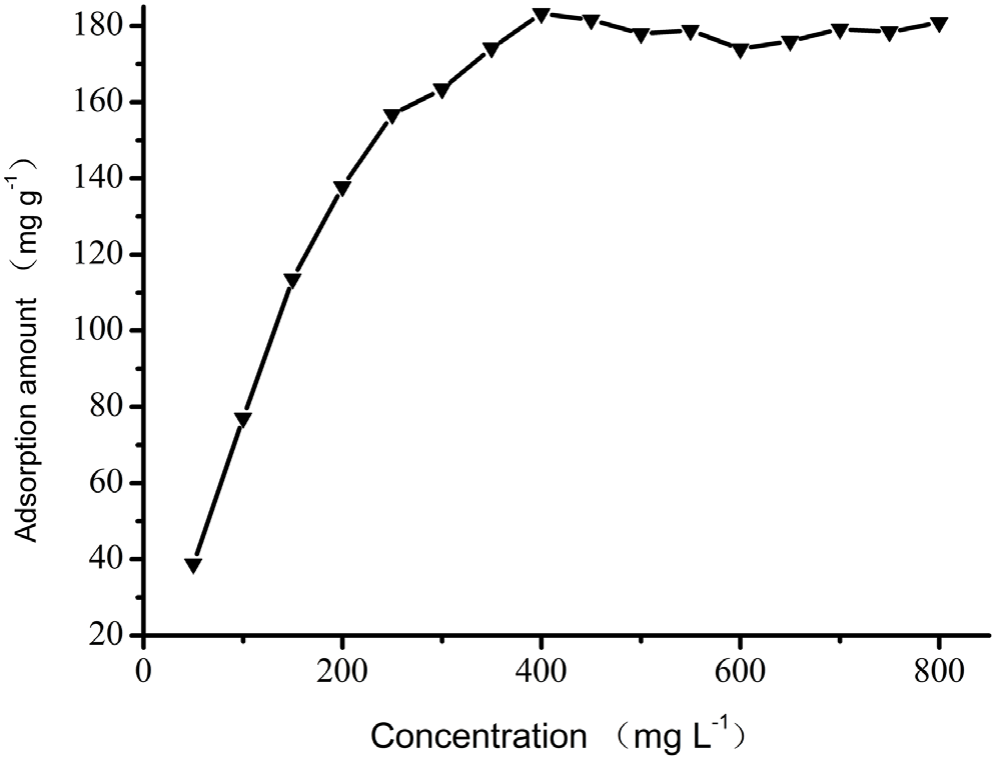
Effect of initial concentration on Orange I adsorption by Fe_3_O_4_-CS.

The effect of the dye concentration on the adsorption amount is shown in Fig. 8. It is clear that the adsorption of Fe_3_O_4_-CS increases with the initial concentration of Orange I in concentrations between 50 and 400 mg L^−1^. The maximum adsorption amount appears at a concentration of 400 mg L^−1^. Between 400 and 800 mg L^−1^, the adsorption amount remains constant at approximately 180 mg g^−1^, and no increase is observed.

**Figure 9:**
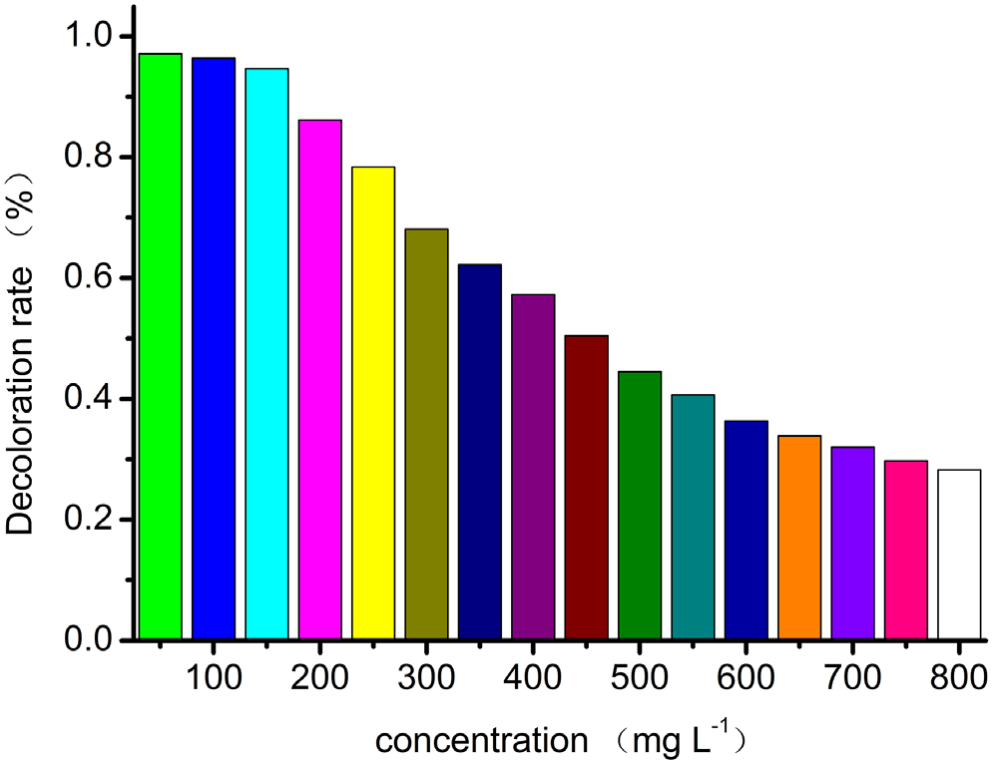
Effect of initial concentration on Orange I decoloration by Fe_3_O_4_-CS.

The decoloration rate is an important parameter in the practical process of wastewater treatment. The decoloration rate of Orange I by Fe_3_O_4_-CS is shown in Fig. 9. It is clear that the decoloration rate decreases with increasing initial concentrations. When the concentration is in the range of 50–150 mg L^−1^, more than 94% of Orange I is removed. The optical concentration range for the decoloration of Orange I is 50–150 mg L^−1^. At this concentration, both the adsorption amount and adsorption efficiency of the Fe_3_O_4_-CS adsorbent are high enough for practical applications.

### 3.4 Effect of contact time

**Figure 10:**
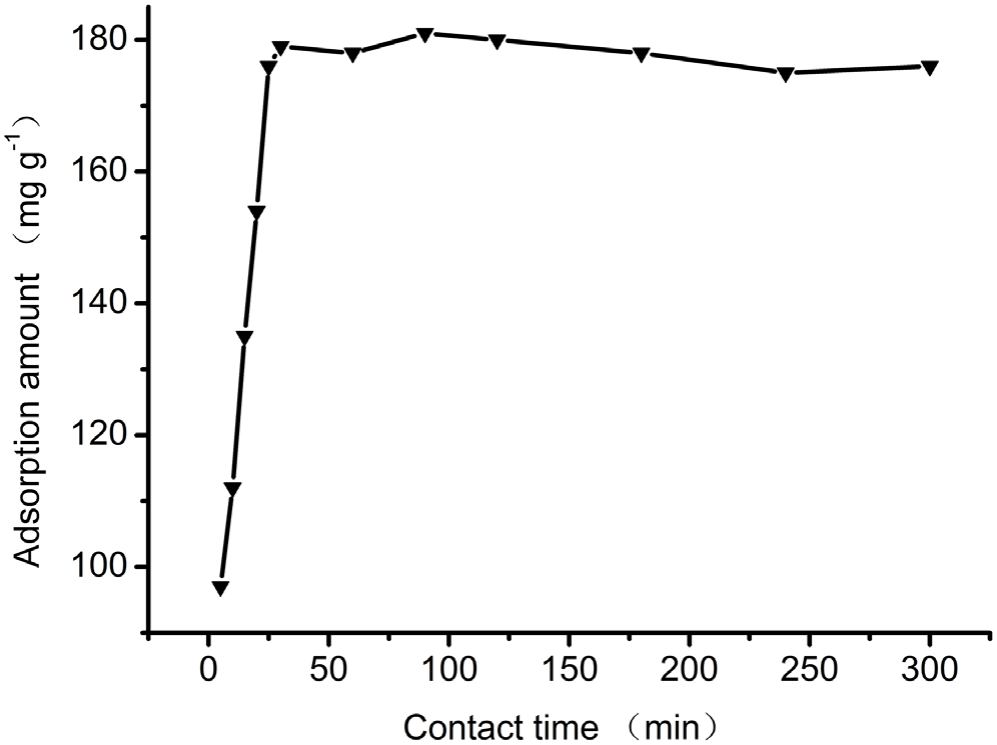
Effect of contact time on Orange I adsorption by Fe_3_O_4_-CS.

The effect of contact time on the adsorption of Orange I is shown in Fig. 10. Adsorption is fastest in the early stages of adsorption process. The adsorbent interacts with dye molecules through electrostatic attraction once the adsorbent is added to the dye solution. After 5 minutes of adsorption, the adsorption amount was over 100 mg g^−1^. This rapid uptake is due to high availability of vacant sites on the surface of the adsorbent [37]. The maximum adsorption amount was observed after 30 minutes. At this point, the vacant sites were all occupied by dye molecules, and saturation was reached. The optical contact time for the adsorption of Orange I was 30 minutes. This short contact time is feasible for practical applications.

### 3.5 Isotherm study

The equilibrium adsorption isotherm is an important parameter in an adsorption system. The Langmuir and Freundlich models were used to describe the equilibrium characteristics of Orange I adsorption onto Fe_3_O_4_-CS.

#### 3.5.1 Langmuir Isotherm

**Figure 11:**
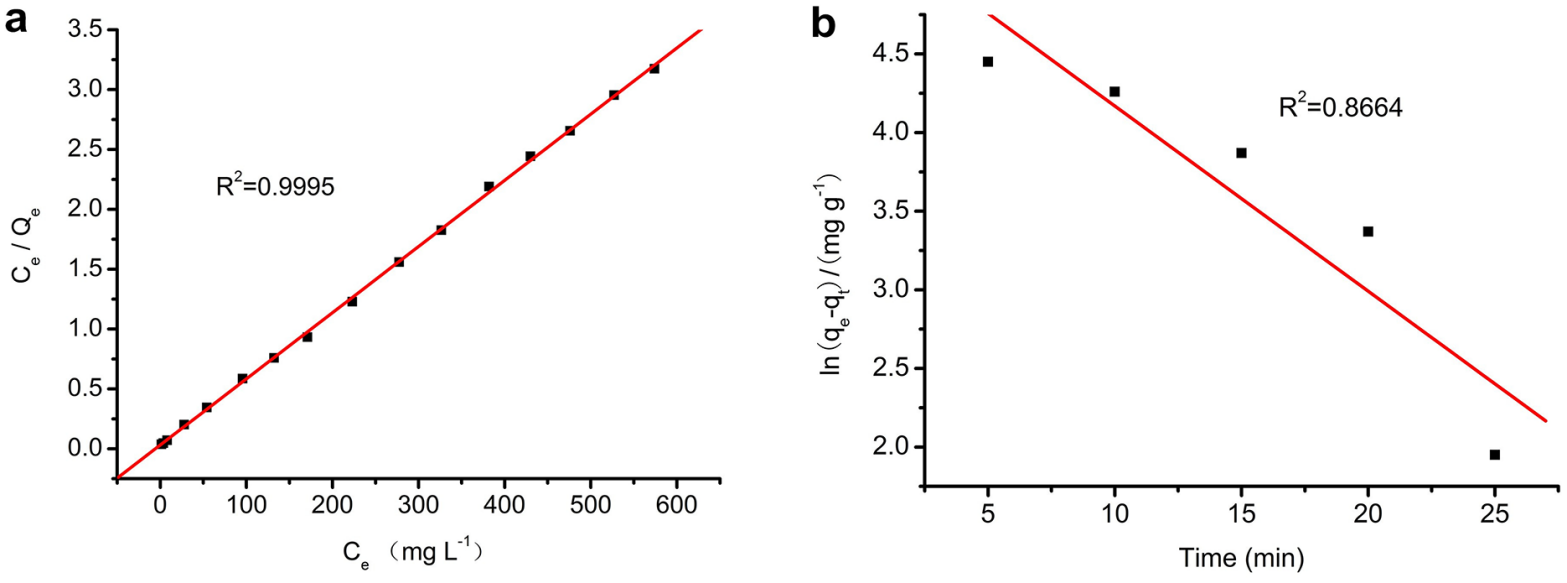
Plots of Langmuir (a) and Freundlich (b) isotherms.

The Langmuir model is based on the assumption of monolayer adsorption without interactions between the adsorbed molecules. The equation can be expressed as:
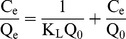
(2)where C_e_ is the equilibrium concentration of Orange I solution (mg L^−1^), Q_e_ is the adsorbed value of Orange I at the equilibrium concentration (mg g^−1^), Q_0_ is the maximum adsorption amount (mg g^−1^), and K_L_ is the Langmuir binding constant. The plot of C_e_/Q_e_ versus C_e_ is a straight line in Fig. 11.(a). The correlation coefficient is R^2^ = 0.9995. The value of Q_0_ obtained from the Langmuir isotherm is 180.8 mg g^−1^, which is perfectly consistent with the experimental data. It also indicates that the adsorption process was mainly monolayer.

#### 3.5.2 Freundlich Isotherm

The Freundlich model is based on the assumption of adsorption on a heterogeneous surface. The equation can be expressed as:
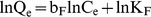
(3)where Q_e_ is the adsorbed value of Orange I at the equilibrium concentration (mg g^−1^), *b*_F_ is a constant describing the adsorption intensity, *K*_F_ is the Freundlich constant, and C_e_ is the equilibrium concentration of Orange I solution (mg L^−1^). Plots of lnQ_e_ versus lnC_e_ are presented in Fig. 11.(b). The correlation coefficient is R^2^ = 0.8174, which indicates that the adsorption isotherm does not fit the Freundlich model very well. The value of *b*_F_ is lower than 1, suggesting a normal Langmuir isotherm [38]. The adsorption isotherm parameters are presented in Table 1.

**Table 1:** Langmuir and Freundlich parameters for adsorption of Orange I by Fe_3_O_4_-CS.

Langmuir model			Freundlich model		
R^2^	Q_0_ (mg g^−1^)	K_L_ (L mg^−1^)	R^2^	K_F_	b_F_
0.9995	180.8	0.1760	0.8174	57.47	0.2025

### 3.6 Adsorption kinetics

To understand the mechanism of adsorption kinetics, pseudo-first-order and pseudo-second-order kinetic models were used to analyze the experimental data. The pseudo-first-order kinetic model can be expressed as [39]:
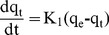
(4)


**Figure 12:**
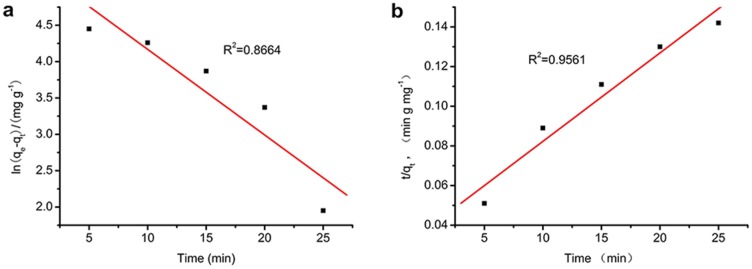
Plots of pseudo-first-order (a) and pseudo-second-order (b) kinetic models.

Under the conditions q_t_ = 0 at t = 0, and q_t_ = q_t_ at t = t, the Equation can be converted into a linear kinetic equation:
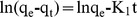
(5)where q_t_ (mg g^−1^) is the amount of Orange I absorbed at time t (min), q_e_ is the amount of adsorbed dye at equilibrium (mg g^−1^), and k_1_ (min^−1^) is the equilibrium rate constant of pseudo-first-order kinetic model. The plot of ln (q_e_–q_t_) through time is presented in Fig. 12.(a).

The pseudo-second order process can be written as follows [40]:
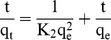
(6)where q_t_ (mg g^−1^) is the amount of Orange I absorbed on the adsorbent at time t (min), q_e_ is the amount of adsorbed dye at equilibrium (mg g^−1^), and k_2_ (g mg^−1 ^min^−1^) is the equilibrium rate constant of pseudo-second-order model. The plot of t/q_t_ over time is presented in Fig. 12.(b). The Orange I pseudo-first-order and pseudo-second-order correlation coefficients are 0.8664 and 0.9561, which illustrates that the pseudo-second-order mechanism offered a better fit than the pseudo-first-order mechanism. The kinetics parameters and rate constants are presented in Table 2.

**Table 2:** Kinetics parameters for the adsorption of Orange I by Fe_3_O_4_-CS.

pseudo-first-order model		pseudo-second-order model		
R^2^	k_1_ (min^−1^)	R^2^	k_2_ (g mg^−1 ^min^−1^)	q_e_ (mg g^−1^)
0.8664	1.178×10^−1^	0.9561	5.277×10^−4^	224.2

## Conclusions

In this study, magnetic Fe_3_O_4_-CS adsorbent was prepared by a one-step method for the adsorptive removal of Orange I from aqueous solutions. High adsorption capacity was achieved through the ionic interactions between protonated amino groups (–NH_3_^+^) of chitosan and sulfonate groups (–SO_3_^−^) of Orange I. The pH, initial concentration and contact time played a significant role in the dye adsorption capacity of Fe_3_O_4_-CS. The maximum adsorption amount reached 183.2 mg g^−1^ at a concentration of 400 mg L^−1^ at pH 2.0. Isotherm modeling revealed that the Langmuir equation could better describe the adsorption of Orange I on the Fe_3_O_4_-CS as compared to Freundlich model and pseudo-second-order kinetic model fitted with experimental data well. The fast uptake and magnetic separation gives the Fe_3_O_4_-CS adsorbent a high potential for effective removal of Orange I in water treatment.
