# Correction: Preparation, Characterization and Application of Magnetic Fe_3_O_4_-CS for the Adsorption of Orange I from Aqueous Solutions

**DOI:** 10.1371/journal.pone.0116073

**Published:** 2014-12-16

**Authors:** The PLOS ONE Staff

Figure 11 is incorrect. Please view the corrected Figure 11 here.

**Figure 11:**
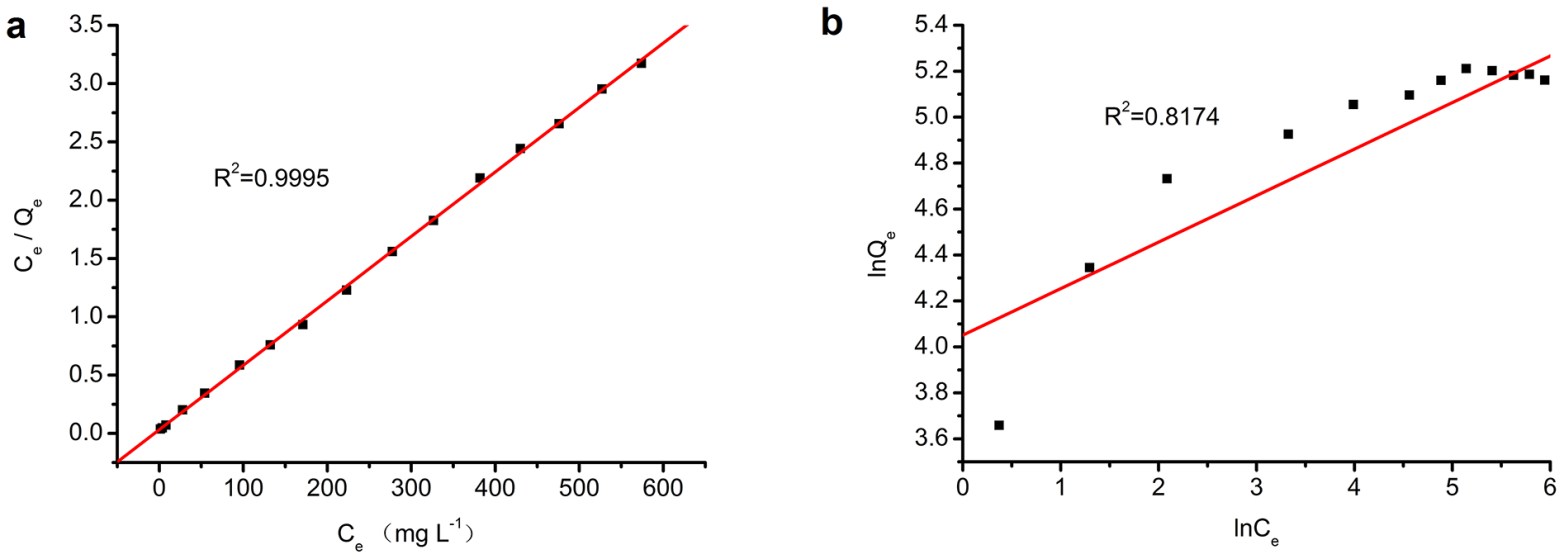
Plots of Langmuir (a) and Freundlich (b) isotherms.
